# Where does the processing of size meet the processing of space?

**DOI:** 10.3758/s13414-024-02979-3

**Published:** 2024-11-12

**Authors:** Peter Wühr, Herbert Heuer

**Affiliations:** 1 Department of Psychology, TU Dortmund University, Dortmund, Germany; 2 Leibniz Research Center for Working Environment and Human Factors (IfADo), Dortmund, Germany

**Keywords:** Additive-factors method, Size-location congruency, SSARC effect, Simon effect, LCA model

## Abstract

Previous studies revealed an S-R compatibility effect between physical stimulus size and response location, with faster left (right) responses to small (large) stimuli, respectively, as compared to the reverse assignments. Here, we investigated the locus of interactions between the processing of size and spatial locations. In Experiment 1, we explored whether stimulus size and stimulus location interact at a perceptual level of processing when responses lack spatiality. The stimuli varied on three feature dimensions (color, size, location), and participants responded vocally to each feature in a separate task. Most importantly, we failed to observe a size-location congruency effect in the color-naming task where S-R compatibility effects were excluded. In Experiment 2, responses to color were spatial, that is, key-presses with the left and right hand. With these responses there was a congruency effect. In addition, we tested the interaction of the size-location compatibility effect with the Simon effect, which is known to originate at the stage of response selection. We observed an interaction between the two effects only with a subsample of participants with slower reaction times (RTs) and a larger size-location compatibility effect in a control condition. Together, the results suggest that the size-location compatibility effect arises at the response selection stage. An extended leaky, competing accumulator model with independent staggered impacts of stimulus size and stimulus location on response selection fits the data of Experiment 2 and specifies how the size-location compatibility effect and the Simon effect can arise during response selection.

**Supplementary information:**

The online version contains supplementary material available at 10.3758/s13414-024-02979-3.

## Introduction

Humans can focus on the behaviorally relevant features of their environment, but their neglect of irrelevant environmental features is less than perfect. This can result in conflicts when currently relevant and irrelevant features call for different actions. Experimentally such conflicts are studied by means of conflict tasks. Some of them, for example, the Stroop task (Stroop, 1935), the Eriksen flanker task (Eriksen & Eriksen, 1974), or the Simon task (Simon & Rudell, 1967; for reviews, see Lu & Proctor, 1995; Proctor & Vu, 2006) are classic and well researched. A more recently discovered conflict task requires rapid responses with the left or right hand to the color of a small or large stimulus. In this task left-hand responses to small stimuli and right-hand responses to large stimuli are faster than left-hand responses to large stimuli and right-hand responses to small stimuli. In analogy to the similar and more extensively studied effect of number magnitude, the SNARC (spatial-numerical association of response codes) effect (Dehaene et al., 1993; see Fischer & Shaki, 2014, for review), the effect of physical size is referred to as the SSARC (spatial-size association of response codes) effect (Ren et al., 2011; Wühr & Seegelke, 2018; Richter & Wühr, 2022). Here we ask whether the facilitating effect of congruent space-size combinations (left-small/right-large) as compared to incongruent combinations (left-large/right-small) originates early during perceptual processing or only later during response selection.

We chose two approaches to answer this question. The first approach is based on the variation of dimensional (spatial) overlap between responses and task-irrelevant stimulus features. The presence or absence of dimensional overlap defines congruent and incongruent conditions and produces the associated performance variations (Kornblum et al., 1990; Kornblum & Lee, 1995). In two experiments we presented stimuli that varied in location, size, and color. In the first experiment we varied the required responses: naming the location, the size, or the color of the stimulus. Crucially, these vocal response sets differ in their spatiality, with responses in the color-naming task having no spatial meaning and thus no spatial dimensional overlap with the irrelevant stimulus features location and size. In the second experiment we mapped stimulus colors to left-hand and right-hand responses, so that responses to colors obtained spatial characteristics. If the SSARC effect originates during response selection, it should appear in response to colors only in the second experiment, not in the first one. If it already appeared in the first experiment, this would suggest that the space and size of the stimuli interact early during perceptual processing.

The second approach is based on the joint effects of the task-irrelevant stimulus features size and location when color is the task-relevant stimulus feature, with two colors being mapped on left-hand and right-hand responses. For the effect of the irrelevant stimulus location, the Simon effect, its origin during response selection is well established (Cespón et al., 2020; Lu & Proctor, 1995). Interactive effects of the irrelevant stimulus size and the irrelevant stimulus location would thus suggest that the effect of irrelevant stimulus size, the SSARC effect, also originates during response selection. Such a conclusion would rely on the additive factors logic for the identification of common and separate processing stages (Sternberg, 1969) and follow the lead of several studies combining the Simon effect with the SNARC effect; with (at least) one exception (Mapelli et al., 2003), these studies found interactive effects (e.g., Gevers et al., 2005; Keus & Schwarz, 2005; Nan et al., 2021; Yan et al., 2021). As the additive factors logic provides only one building block of a firm basis for theoretical conclusions (Pachella, 1974; Sternberg, 1998; Taylor, 1976), we complement it with a sequential-sampling model that accounts for the combined Simon and SSARC effects in terms of response selection (cf. Pieters, 1983, who contrasts stage analysis and distribution analysis as functional and structural approaches, respectively).

Previous studies revealed evidence suggesting a special role of the two hands as effectors for the origin of the SSARC effect. For example, when exploring the spatial coding of the responses that is relevant for the SSARC effect, Seegelke et al. (2023) observed that the SSARC effect was restricted to tasks in which participants had to choose between the two hands for responding, whereas the SSARC effect did not occur when participants had to choose between two fingers of the same hand for responding. This finding stands in striking contrast to other compatibility or congruency effects involving spatial responses, such as the Simon or the SNARC effect, that have been observed both when response selection requires a choice between two hands, and when response selection requires a choice between two fingers of the same hand (e.g., Heister et al., 1987; Priftis et al., 2006). In addition, Wühr et al. (2024) demonstrated that asymmetries in hand strength correlate positively with the size of the SSARC effect. Based on this and other observations, Wühr et al. suggested that strength differences between the dominant and the non-dominant hand might contribute to the origin of the SSARC effect. In particular, because the dominant hand is stronger than the non-dominant hand (cf. Bohannon, 2003, for review), people might learn to grasp larger (and heavier) objects more often with the dominant rather than with the non-dominant hand, and this habit might eventually lead to the SSARC effect. The reported findings indicating a special role of the hands for the (origin of the) SSARC effect may suggest a later functional locus of the SSARC effect, such as response selection. On the other hand, however, these findings do not preclude the possibility of interactions between the processing of stimulus size and stimulus location at earlier, perceptual, levels of processing as well.

## Experiment 1

In Experiment 1 we varied stimulus features on the three dimensions color, size, and location. Participants responded vocally to each stimulus dimension in a separate task: they named stimulus color in the color-naming task, stimulus size in the size-naming task, and stimulus location in the location-naming task. These different vocal responses differ with respect to their spatiality.

Naming colors has no apparent spatial meaning. Thus, if space-size integration was bound to response selection, there should be no performance differences between congruent (left-small/right-large) and incongruent (left-large/right-small) irrelevant stimulus features. In contrast, if space-size integration occurred during perceptual processing, the perception of small stimuli should be facilitated by a left location as compared with a right location, and the perception of large stimuli should be facilitated by a right location as compared with a left location. Such perceptual facilitations and/or inhibitions should result in faster and slower responses even when these have no spatial characteristics such as the naming of colors. Vice versa, observing performance differences between stimuli with congruent and incongruent irrelevant features would suggest integration of size and location processing during perception.

Obviously, naming locations is a response with spatial characteristics. Thus, a regular SSARC effect would be expected in the location-naming task (e.g., Wühr et al., 2024), with better performance for small-“left” and large-“right” than for small-“right” and large-“left,” even when size and space interact during response selection rather than during perceptual processing. Although naming sizes has no spatial meaning at first glance, there may be an indirect spatiality based on experienced associations (cf. Wühr et al., 2024). Thus, a reciprocal SSARC effect seems possible in the size-naming task with better performance for left-“small” and right-“large” than for right-“small” and left-“large,” even when size-space interactions are bound to response selection. However, a previous study showed that regular SSARC effects, in a task with size stimuli and vocal location responses, and reciprocal SSARC effects, in a task with location stimuli and vocal size responses, are asymmetrical (Richter & Wühr, 2023). The regular SSARC effect was much bigger than the reciprocal SSARC effect, and the reciprocal effect, which appeared only in the highest quartile of the reaction time (RT) distributions, even disappeared when a small group of participants with long (outlier) RTs were excluded from data analysis (Richter & Wühr, 2023).

## Method

### Openness and transparency

We report how we determined our sample size, data exclusion, all manipulations, and all measures in the study. The data from both experiments are available at the Mendeley repository (10.17632/3v79sh9njf.1). The local Ethics Committee at TU Dortmund University (2018-09) approved the experimental protocol for both experiments.

Experiment 1 was preregistered at the Open Science Framework (OSF) at: 10.17605/OSF.IO/GCXNM, on 7 May 2022, before data collection began.

### Participants

Our previous studies revealed size-location correspondence effects with an average effect size of ${\eta }_{p}^{2}$ = .15 (e.g., Wühr & Richter, 2022; Wühr & Seegelke, 2018). Given this value, we decided to collect data from at least 72 participants, because this sample size allows us to detect a 2 x 3 interaction of moderate size (i.e., ${\eta }_{p}^{2}$ = .10) with high power (1-beta = .95) at the standard .05 alpha error probability according to MorePower (Campbell & Thompson, 2012).

The actual sample consisted of 78 participants, mostly students of TU Dortmund University. The 47 female and 31 male participants had an average age of 21.44 years (*SD* =3.51). According to self-report, all participants had normal or corrected-to-normal vision. They gave their informed consent prior to participation and received either course credit or a payment of 12 Euro.

Participants filled in a modified version of the Edinburgh Handedness Inventory (Oldfield, 1971). On a five-point rating scale they indicated how often they performed each of ten activities with the left or right hand. The answers were coded as -2 (always left), -1 (mostly left), 0 (sometimes left, sometimes right), 1 (mostly right), and 2 (always right). The sum was used to compute a handedness score as (sum/20)*100, which ranges from -100 to +100. Participants scoring above 40 were classified as right-handers, participants scoring between -40 and 40 were classified as ambidexters, and participants scoring below -40 were classified as left-handers. Six participants were left-handers (mean score = -69.17), two participants were ambidexters (mean score = 32.50), and 70 participants were right-handers (mean score = 78.43). Notably, the score-based handedness classifications matched participants’ self-classifications in 76 cases; exceptions were the two ambidexters, who had classified themselves as right-handers.

### Apparatus and stimuli

Participants sat in front of a 24-in. monitor with a viewing distance of approximately 50 cm. We used the software E-Prime 3.0 (Psychology Software Tools; Sharpsburg, PA, USA) to control the presentation of stimuli and register responses (i.e., key pressed, RT). A plus sign (Courier font, size 18 pt) served as a fixation point. The imperative stimulus was a filled square that varied on three dimensions: color, size, and horizontal position. In particular, the square was either red (RGB 254,0,0) or green (RGB 0,175,80), either small (i.e., side length of 2 cm) or large (i.e., side length of 4 cm), and it appeared either 4 cm to the left or 4 cm to the right of fixation. The fixation point and the imperative stimulus were presented on a gray background (RGB = 192,192,192). Participants responded vocally. A microphone was placed directly in front of them and was connected to the voice-key of the Chronos console (Psychology Software Tools; Sharpsburg, PA, USA). In addition to RT each vocal response was recorded and stored on the computer.

### Procedure

There were three choice-response tasks that differed with regard to the relevant stimulus feature and the corresponding (vocal) responses. In the color-naming task, participants had to name the color (green or red) of the imperative stimulus as quickly as possible. In the location-naming task, participants had to name the location (left or right) of the imperative stimulus as quickly as possible. Finally, in the size-naming task, participants had to name the size (small or large) of the imperative stimulus as quickly as possible. Each task was performed for one training block of 16 trials and two experimental blocks of 48 trials each. Trials occurred in random order within each block. Each trial started with a blank screen for 500 ms. Next, the fixation point was presented for 400 or 600 ms at screen center, with both durations occurring equally often within a block, followed by the imperative stimulus until a response was made, or for a maximum of 2,000 ms. Each response was followed by an empty screen for 1,500 ms; when no response was detected, a corresponding message (“No response detected”) was shown during this inter-trial interval. The program could not determine the accuracy of the spoken responses, and therefore no feedback about response accuracy was provided.

The six possible orders of the three tasks were balanced across participants. Participants could take a break between blocks (or continue immediately with the next one), and between tasks, which had to be started by the experimenter. The experiment took about 30 min. For each task, the experimenter stayed in the laboratory for the practice block and left the laboratory before participants started the first experimental block of trials.

### Design and data analysis

The raw data obtained in Experiment 1 can be found at the Mendeley repository (10.17632/3v79sh9njf.1). The experiment had a three-factorial 3 × 2 × 2 design with Task (color naming, size naming, location naming), Stimulus Size (small, large), and Stimulus Location (left, right) as within-subjects variables.^1^ Dependent variables were the RTs of correct vocal responses and the percentages of incorrect responses. We excluded the training blocks, the first trial in each experimental block, and trials in which RTs were below 100 ms or above 1,500 ms from data analysis. We also applied the Tukey criterion (i.e., Quartile_25_ – 1.5 × IQR; Quartile_75_ + 1.5 × IQR) for each task to discover outlier values in overall RT and overall error percentages. The maximal error percentage for each participant in each task was below 5%, which we considered acceptable. Several participants showed outlier RTs in one or two tasks, but we only excluded two participants who showed outlier RTs in each of the three tasks.

## Results

### Reaction times (RTs)

A three-factorial ANOVA with the within-participant factors Task, Stimulus Size, and Stimulus Location revealed a significant main effect of Task, *F*(2,150) = 100.894, *MSE* = 3,849.9, *p* < .001, ${\eta }_{p}^{2}$ = .57: RTs in the location-naming task were shorter (*M* = 387 ms, SD = 51 ms) than in the color-naming task (*M* = 450 ms, SD = 55 ms) and the size-naming task (*M* = 448 ms, SD = 55 ms). Crucially, the three-way interaction was significant, *F*(2,150) = 6.306, *MSE* = 328.8, *p* = .002, ${\eta }_{p}^{2}$ = .078, suggesting different patterns of results for the three tasks. Therefore, we conducted three separate two-way ANOVAs with Stimulus Size and Stimulus Location as within-participant factors.^2^

**Fig. 1:**
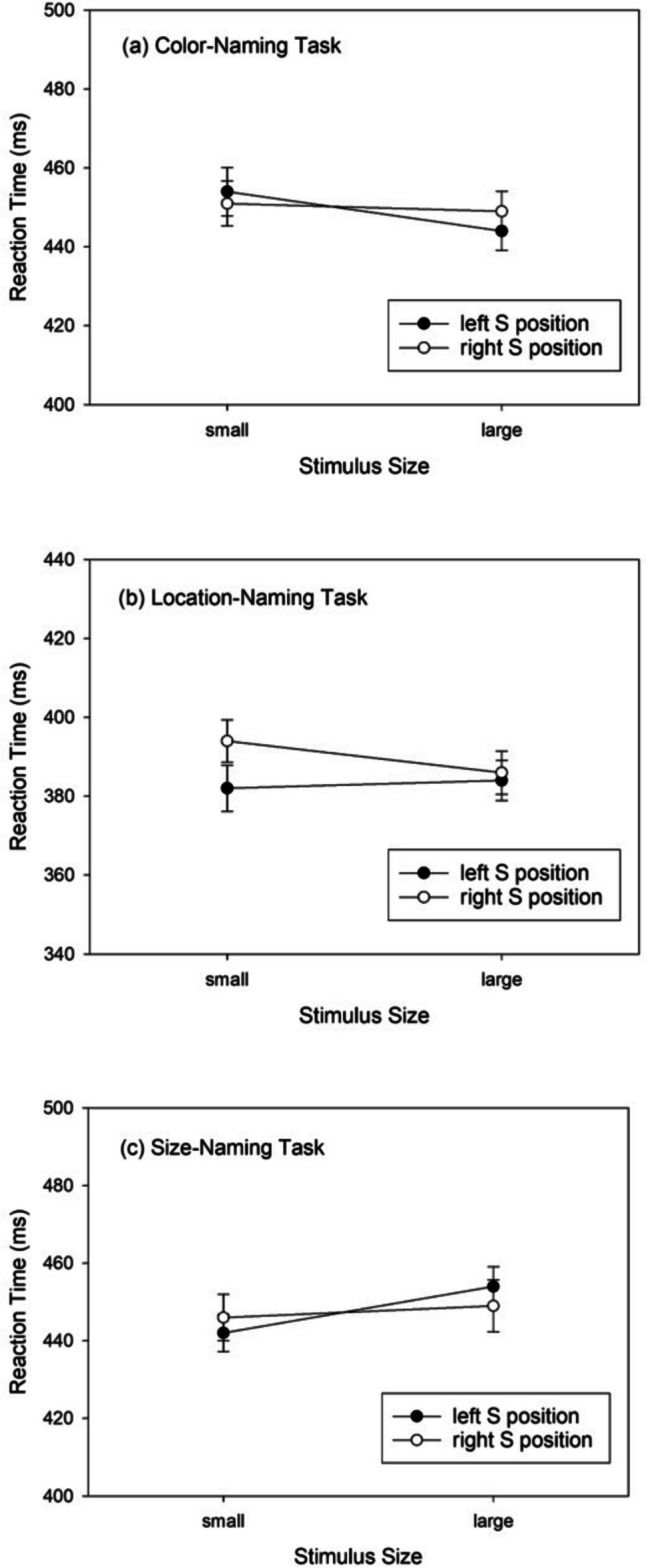
Reaction times (RTs) observed in Experiment 1 as a function of stimulus size, stimulus location, and task. Error bars show 95% confidence intervals for within-subjects designs (Cousineau, 2017)

For the *color*-naming task, the mean RTs are shown in Fig. 1a. RTs for large stimuli were shorter than for small stimuli. The two-way ANOVA revealed a significant main effect of stimulus size, *F*(1,75) = 7.130, *MSE* = 378, *p* = .009, ${\eta }_{p}^{2}$ = .087. Numerically, RTs for small stimuli were shorter in the right location (*M* = 451 ms, *SD* = 54) than in the left location (*M* = 454 ms, *SD* = 59), and for large stimuli the effect of location was reversed (*M* = 444 ms, *SD* = 53, for left location; *M* = 449 ms, *SD* = 63, for right location). This pattern is the opposite of what is found in the SSARC effect (or a negative SSARC effect of -4 ms), where RTs to small stimuli are shorter in the left than in the right location and to large stimuli in the right than in the left location. However, both the main effect of stimulus location, *F*(1,75) = 0.425, *MSE* = 420, *p* = .517, ${\eta }_{p}^{2}$ = .006, and the two-way interaction, *F*(1,75) = 3.442, *MSE* = 414, *p* = .067, ${\eta }_{p}^{2}$ ^2^ = .044, were not significant. Thus, for color-naming there is no indication of space-size integration.

For the *location*-naming task the RT means are shown in Fig. 1b. The main effect of stimulus size, *F*(1,75) = 1.910, *MSE* = 300, *p* = .171, ${\eta }_{p}^{2}$ = .025, was not significant, but there was a significant main effect of stimulus location, *F*(1,75) = 6.530, *MSE* = 606, *p* = .013, ${\eta }_{p}^{2}$ = .080: RTs to left stimuli were shorter than RTs to right stimuli. The two-way interaction, *F*(1,75) = 6.790, *MSE* = 287, *p* = .011, ${\eta }_{p}^{2}$ = .083, was also significant: RTs to small-left stimuli (*M* = 382 ms, *SD* = 54) were shorter than to small-right stimuli (*M* = 394 ms, *SD* = 54), *t*(75) = -3.424, *p* = .001, *d* = -0.393, whereas the difference between large-left (*M* = 384 ms, *SD* = 55) and large-right stimuli (*M* = 386 ms, *SD* = 51), *t*(75) = -0.661, *p* = .510, *d* = -0.076, was not significant. This is the interaction pattern expected when a benefit of small-left and large-right stimuli as compared with small-right and large-left stimuli is superposed by overall faster RTs to left stimuli. It amounts to an SSARC effect of 5 ms.

Finally, for the *size*-naming task the RT means are shown in Fig. 1c. There was a significant main effect of stimulus size, *F*(1,75) = 5.841, *MSE* =680, *p* = .018, ${\eta }_{p}^{2}$ = .072, which was opposite to the effect of stimulus size for color naming in that RTs to small stimuli were shorter than to large stimuli. The main effect of stimulus location, *F*(1,75) = 0.194, *MSE* = 343.9, *p* = .661, ${\eta }_{p}^{2}$ = .003, was not significant. A significant two-way interaction, *F*(1,75) = 5.387, *MSE* = 264.2, *p* = .023, ${\eta }_{p}^{2}$ = .067, reflected numerically shorter RTs for small-left stimuli (*M* = 442 ms, *SD* = 56) relative to small-right stimuli (*M* = 446 ms, *SD* = 55), *t*(75) = -1.336, *p* = .185, *d* = -0.153, and numerically shorter RTs for large-right stimuli (*M* = 449 ms, *SD* = 62) relative to large-left stimuli (*M* = 454 ms, *SD* = 58), *t*(75) = 1.702, *p* = .093, *d* = 0.195. This is the interaction pattern that corresponds to the (reciprocal) SSARC effect of 4 ms.

### Error percentages

The mean error percentages are reported in Table 1. Errors were very rare in each condition and task. In fact, zero errors (across all four conditions) was the most frequent result in each task (color-naming task: 59%; location-naming task: 54%, size-naming task: 58% of all participants). Hence, the mean error rate is not representative for the performance of the majority of participants; the median error rate in each task was actually zero. Therefore, we refrained from further analyzing error percentages in this experiment.

**Table 1:** Error percentages observed in Experiment 1 as a function of Task, Stimulus Size, and Stimulus Location (standard deviations are given in parentheses)

	Small S		Large S	
Task	Left S	Right S	Left S	Right S
Color-Naming Task	0.439 (1.287)	0.603 (1.476)	0.439 (1.287)	0.822 (2.154)
Location-Naming Task	0.274 (1.040)	0.713 (1.580)	0.987 (2.026)	0.548 (1.573)
Size-Naming Task	0.493 (1.516)	0.658 (1.807)	1.151 (2.317)	0.603 (1.625)

S = stimulus

### Effects of task order on RTs

In an additional analysis, which was not pre-registered, we explored whether task order modulated the size-location congruency effect in the different tasks. The orders of the three tasks had been counterbalanced across participants, with 13 participants per each of the six orders. The exploratory analysis was motivated by the idea that an irrelevant stimulus feature might have a stronger impact in a given task when it had been the relevant feature in the preceding task as compared with a neutral feature having been relevant. For example, the reciprocal SSARC effect in the size-naming task might be larger when this task was preceded by the location-naming task than when preceded by the color-naming task. We tested for the effects of task order in separate 2 (stimulus size) x 2 (stimulus location) x 6 (task order) ANOVAs for each task with RTs as the dependent variable. The results revealed no effects of task order whatsoever. For each task, the ANOVA revealed a non-significant two-way interaction of stimulus size and task order, all *F*s(5,70) < 1.00, all *p*s > .600, a non-significant two-way interaction of stimulus location and task order, all *F*s(5,70) < 1.20, all *p*s > .300, and a non-significant three-way interaction, all *F*s(5,70) < 1.10, all *p*s > .300. Although the power of these analyses is relatively low, the results provide no hint of an effect of task order on the RT results.

### Distributional analyses

**Fig. 2:**
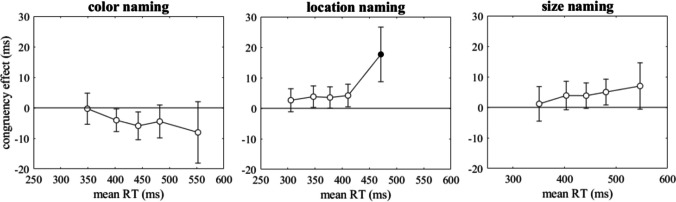
Differences between size-location incongruent and congruent conditions at five quantiles of reaction time (RT) distributions as a function of the mean quantiles in the two conditions (delta plots) for the three tasks of Experiment 1. Error bars show 95% confidence intervals. Filled circles indicate significant differences from zero (after Bonferroni-Holm correction)

In a second additional analysis, which was not pre-registered, we analyzed the dynamics of the size-location congruency effects in the different tasks. For each task we computed five quantiles (.1, .3, .5, .7, .9) of the distributions of RTs of correct responses in size-location congruent and incongruent conditions. Figure 2 shows the delta plots, that is, the differences between incongruent and congruent conditions at each quantile as a function of the mean quantiles in the two conditions. Incongruent and congruent conditions were compared by means of t-tests for repeated measures at each quantile. Significant tests (after Bonferroni-Holm correction) are indicated by filled circles, whereas non-significant effects of size-location congruency are indicated by open circles.

For the color-naming task, the size-location congruency effect was negative throughout the whole range of RTs, but for no quantile it was statistically significant. This corresponds to the findings for mean RT. For the location-naming task, a significant size-location congruency effect was observed at long RTs, whereas at short RTs it amounted essentially to zero. For the size-naming task the (reciprocal) congruency effect did not reach statistical significance at any of the five quantiles. This casts some doubts on the robustness of the (reciprocal) congruency effect of 4 ms, which was statistically significant for mean RTs.

## Discussion

In Experiment 1 we varied stimulus features on three dimensions (color, size, location), and participants had to verbally respond to each feature dimension in a separate task. The most interesting task was color-naming where interactions during concurrent perceptual processing of size and location were possible, but not during response selection because color names have no spatial characteristics so that there is no dimensional overlap between stimulus and response features. Crucially, for color naming there were no size-location congruency effects in RTs (and accuracy), that is, better performance with small stimuli in left than in right locations and large stimuli in right than in left locations. Rather, the results showed numerical trends in the opposite direction: Responses were slower to the small-left than to the small-right stimuli and to the large-right than to the large-left stimuli, and accuracy was generally higher for left as compared to right stimuli.

Location-naming and size-naming differed from color-naming with respect to the spatiality of responses. Thus, in the location-naming task, we observed a regular SSARC effect: The vocal “left“ response was faster than the vocal “right“ response to the small stimulus, while there was no difference in responses to the large stimulus. This result confirms that irrelevant stimulus size does not only produce SSARC effects in manual responses (e.g., Wühr & Seegelke, 2018), but also in vocal responses (Wühr et al., 2024). As is typical for SSARC effects (e.g., Heuer et al., 2023; Richter & Wühr, 2023), the effect was essentially absent at short RTs and increased as RTs became longer. The size of the vocal effect observed in Experiment 1 (${\eta }_{p}^{2}= .083$) was, however, considerably smaller than the effect of irrelevant size with manual responses (${\eta }_{p}^{2}$ = .356; Wühr & Seegelke, 2018), and the effect of relevant size with vocal responses (${\eta }_{p}^{2}$ = .333; Wühr et al., 2024). A possible reason for the different effect sizes relates to the fact that stimuli varied on three feature dimensions in the present experiment, whereas stimuli varied on only two feature dimensions (color, size) in the previous studies. Hence, it is possible that additional variation of stimulus location has decreased the SSARC effect.

In the size-naming task, we observed a reciprocal SSARC effect in vocal responses. The observation of this reciprocal SSARC effect is somewhat surprising because we failed to obtain a reciprocal SSARC effect in a previous study (Richter & Wühr, 2023). However, it was only small and may not be a robust phenomenon, as in the analyses of the quantiles of the RT distributions it failed to reach statistical significance. Yet, there were several methodological differences between the studies that are discussed in the General discussion.

## Experiment 2

In Experiment 2 stimuli again differed in color, size, and location, but participants responded to colors with left-hand or right-hand key-presses rather than with naming them. Thus, responses had spatial characteristics, and different from the color-naming task of Experiment 1, size-location interactions should appear in the format of a manual SSARC effect, that is, with faster left-hand responses to small than to large stimuli and faster right-hand responses to large than to small stimuli. This contrasts with the findings with the color-naming task of Experiment 1 and would be consistent with response selection as the origin of size-space interactions.

In Experiment 2 we also followed the second approach to answer the question whether size and space interact during early perceptual processes or later processes of response selection. Following the additive-factors logic of Sternberg (1969, 1998), we ask for interactions between the effects of irrelevant size, the SSARC effect, and irrelevant stimulus location, the Simon effect. Given the evidence that the Simon effect arises during response selection, an interaction of irrelevant location and irrelevant size would suggest that the SSARC effect also originates during response selection.

The notion that the Simon effect originates during response selection rather than during perceptual processing is supported by interactions of irrelevant stimulus location with factors that most likely affect response selection, and additive effects with factors that most likely affect perceptual processing. For example, the Simon effect is larger when participants have to select between responses with two fingers of the same hand than between responses with fingers of different hands (e.g., Buckolz et al., 1996; Wühr & Heuer, 2018). On the other hand, the quality of a visual stimulus does not interact with the Simon effect induced by the location of an irrelevant tone (e.g., Acosta & Simon, 1976; Simon, 1982; Simon & Pouraghabagher, 1978).

The results of electrophysiological studies, particularly those of the lateralized readiness potential (LRP), converge with those of behavioral studies. The LRP indicates the relative increase of activation in primary motor cortex that precedes the movement of the left or right hand or fingers of the left or right hand (cf. Leuthold, 2011). Studies of the Simon effect reported an LRP reflecting activation of the hand or finger on the spatially congruent side that occurs both in congruent and in incongruent trials (e.g., Stürmer et al., 2002; Valle-Inclán, 1996; Van der Lubbe et al., 2001; see, Leuthold, 2011, for review). That means that, in incongruent trials, the LRP indicates an automatic activation of the incorrect, spatially congruent response, before the correct response is eventually activated and executed. This automatic activation sometimes gives rise to electromyographic activity without an overt incorrect response (e.g., Burle et al., 2002).

The conclusions based on the additive-factors logic can be doubted, and the rationale is silent with respect to the actual processing, for example, during response selection. Therefore, we added a model-based analysis which made use of sequential-sampling models, specifically extended Leaky, Competing Accumulator (LCA) models (cf. Usher & McCelland, 2001) as they had been separately applied both to the Simon and the SSARC effect (Heuer et al., 2023). Similar to how Mahani et al. (2019) used the Diffusion Model for Conflict tasks (DMC) (Ulrich et al., 2015) to model the effects of two irrelevant stimulus features, we complemented the relevant input of an LCA model with two rather than one irrelevant input related to irrelevant location and size of the stimulus, respectively. Thus, the purpose of the model was to test specifically whether the combined effects of irrelevant stimulus location and size can be accounted for by separate impacts of these features on response selection (additional modeling results are reported in Appendix D in the Online Supplemental Material (OSM)).

Here we briefly describe the basics of LCA models and of our extensions. Details are provided in Appendix A (OSM). For a binary decision the correct and error responses are represented by two response codes which cumulate incremental activations until activation of one of them reaches a threshold. The incremental activation per unit of time is the sum of external, stimulus-related inputs, self-inhibition, lateral inhibition, and noise. Self-inhibition shapes the negative acceleration of response-code activation and its final asymptote, lateral inhibition increases the activation difference between the two response codes, and the noise produces errors and variability of the number of units of time until the threshold is reached. To this time a residual time is added to take the durations of stimulus identification and response production into account.

In the basic LCA model the external input to the correct response code is constant across time. The external input to the error response code is one minus the external input to the correct response code. Setting the sum of external inputs to the two response codes to one amounts to defining a scale for a subset of model parameters, as is required by essentially all sequential-sampling models (cf. Donkin et al., 2009).

To account for congruency effects, we made additions to the external, stimulus-related inputs. Similar to a computational model of Zorzi and Umiltá (1995) and an extension of the diffusion model by Ulrich et al. (2015), we added to the time-constant “controlled input” a time-varying “automatic input,” related to the task-relevant and task-irrelevant stimulus features, respectively. For the automatic input we assumed an exponential decline and a variable temporal offset to the controlled input (Heuer et al., 2023; Wühr & Heuer, 2018). For example, in the Simon task processing of the (task-irrelevant) stimulus position leads the processing of the task-relevant stimulus color (Cespón et al., 2020; Zorzi & Umiltá, 1995), whereas processing of the task-irrelevant stimulus size lags with regard to the processing of the task-relevant stimulus color (Heuer et al., 2023). The staggered onset of response activation by different stimulus features is a distinctive attribute of the model.

**Fig. 3:**
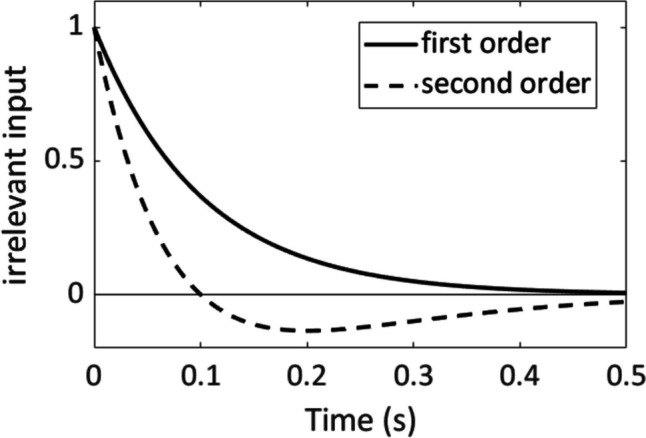
Time course of the automatic input related to stimulus size and location in the first-order and the second-order model

Here we explored two extended LCA models which differed in the time course of the decay of the automatic inputs, as illustrated in Fig. 3. In the “first-order model” the influence of the irrelevant features declined exponentially, as in the models described by Heuer et al. (2023). Exponential decline is the response of a first-order high-pass filter to a step input. In the “second-order model” we used the response of a second-order high-pass filter, which is more similar to the time course assumed in the DMC model of Ulrich et al. (2015). Specifically, the instantaneous influence of an irrelevant feature is not only allowed to decline, but also to reverse (becoming negative). This time course is an approximation of an exponential decline that in several trials, specifically trials that follow incongruent ones, is superimposed by an activation of the incongruent response, which can result in an inverted total effect of the irrelevant feature (cf. Heuer & Wühr, 2024; Tagliabue et al., 2000). Whereas the precise modeling of the differences between congruency effects following congruent and incongruent trials requires additional model parameters, the present approximation for mixtures of congruency effects after congruent and incongruent trials does not.

Until now, the SSARC effect has always been investigated in experiments in which stimulus size was either the relevant stimulus feature or the only irrelevant stimulus feature (e.g., Richter & Wühr, 2022; Seegelke et al., 2023; Wühr et al., 2024; Wühr & Seegelke, 2018). It is therefore unclear how variation of a second irrelevant stimulus feature (location) might influence the SSARC effect. It is possible that this weakens the processing of stimulus size and therefore decreases the SSARC effect. To address this problem, we added a control condition with central stimulus presentation (as in previous experiments), before administering the critical conditions with lateral stimulus presentation. Adding this control condition allowed us to evaluate the effects of an additional variation of stimulus location on the SSARC effect. In addition, the control condition allowed us to investigate whether the interaction between Simon and SSARC effects in the main experiment depends on the size of the regular SSARC effect in the control condition.

## Method^3^

### Participants

A previous study on the functional locus of the SNARC effect observed a significant two-way interaction between the SNARC effect and the Simon effect with ${\eta }_{p}^{2}$ = 0.13 (Yan et al., 2021). Since we are lacking studies on statistical interactions between SSARC and Simon effects, we used this effect size as a reference. According to MorePower (Campbell & Thompson, 2012), a sample size of 90 participants is required to detect a 2 x 2 (or a 2 x 2 x 2) interaction of this size (i.e., ${\eta }_{p}^{2}$ = .13) with high power (1-beta = .95) at the standard .05 alpha error probability.

Our sample consisted of 63 female and 27 male participants, mostly students at TU Dortmund University, with an average age of 22.14 years (*SD* = 3.68). All participants declared having normal or corrected-to-normal vision, and 85 of them described themselves as right-handed. Participants gave their informed consent prior to participation and received either course credit or a coffee voucher (worth = € 5.00).

### Apparatus and stimuli

We used the same apparatus and software as in Experiment 1. A plus sign (Courier font, size 18 pt) served as a fixation point. The imperative stimulus was a filled circle that varied in three dimensions: color, size, and horizontal position. In particular, the circle was either red (RGB 180,20,20) or green (RGB 20,180,80), either small (i.e., diameter of 2 cm) or large (i.e. diameter of 4 cm), and it could appear at screen center, or 8 cm to the left or right of the center of the fixation point. The fixation point and the imperative stimulus were presented on a gray background (RGB = 192,192,192). Participants responded manually by pressing the left Control key (at the left margin of the keyboard) or the right Enter key (at the right margin of the keyboard) on a regular Windows keyboard.

### Procedure

The experiment consisted of two parts. In both of them, participants had to respond to the color of the imperative stimulus. Half of the participants pressed the left key in response to the green stimulus and the right key in response to the red stimulus; the other participants used the opposite S-R mapping. The two parts of the experiment differed with regard to stimulus location. In the first part, which consisted of a practice block (16 trials) and one test block (48 trials), the stimulus was always presented at screen center. In the second part, which consisted of four test blocks with 48 trials each, the imperative stimulus was presented either to the left or right of fixation. The eight types of trials (2 colors x 2 sizes x 2 locations x 6 repetitions) were randomized with the constraint of equal frequencies within each block.^4^

Each trial started with a blank screen of 500 ms. Next, the fixation point was presented for 400 or 600 ms at screen center, with both durations occurring equally often within a block, followed by the imperative stimulus, which remained on until a response was made or for a maximum of 2,000 ms. Each correct response was followed by an inter-trial interval of 1,000 ms with an empty screen. If an incorrect key was pressed, or if no response was detected, a corresponding error message was shown during the inter-trial interval.

Participants could take a break between blocks, or continue immediately with the next one. The experiment took about 30 min. The experimenter stayed in the laboratory for the practice block, and left the laboratory before participants started the first test block.

### Design and data analysis

The raw data obtained in Experiment 2 can be found at the Mendeley repository (10.17632/3v79sh9njf.1). The first part of the experiment with central stimulus location had a one-factorial design with Size Congruency (SSARC effect) as a within-participant variable. The second part with peripheral stimulus locations had a two-factorial 2 × 2 design with Location Congruency (Simon effect) and Size Congruency (SSARC effect) as within-participant variables.^5^ Dependent variables were the RTs of correct key-press responses and the percentages of incorrect responses.

We excluded the training blocks, the first trial in each experimental block, and trials in which RTs were below 100 ms or above 1,500 ms from data analysis. We also applied the Tukey criterion (i.e., Quartile_25_ - 1.5 × IQR; Quartile_75_ + 1.5 × IQR) to discover outlier values in overall RT and in overall error percentages for each task. An outlier screening revealed no outliers in overall error percentage (*PE*_max_ = 10.417%), and three outliers in overall RT (*M*_RT_ = 424 ms; *SD*_RT_ = 62; *RT*_max_ = 662 ms). Because the participants with long RTs had relatively small error percentages (i.e. < 6%), and long RTs therefore reflect a careful response criterion rather than poor overall performance, we decided not to exclude these participants from analysis.

For the model-based analysis we pooled the data of all participants for each of the four conditions of the second part of the experiment by linear transformations as described by Sternberg (2024). Specifically, the reaction time ${x}_{ij}$ in trial *j* of participant *i* in any of the four conditions was transformed into ${y}_{ij}={m}_{.}+\left({x}_{ij}-{m}_{i}\right)\frac{{q}_{.}}{{q}_{i}}$, where ${m}_{i}$ is the mean of participant *i* and ${m}_{.}$ is the mean of these individual means. Similarly, ${q}_{i}$ is a robust scale estimator for participant *i* and ${q}_{.}$ is the mean of the individual scale estimators. The scale estimator is based on an order statistic of the absolute differences between observations (Croux & Rousseeuw, 1992; Rousseeuw & Croux, 1993). According to Sternberg (2024), this method of combining individual RT distributions is more accurate in estimating the average of the individual distributions than other and more established methods such as vincentizing (Ratcliff, 1979).

From the pooled data, 4,211–4,229 trials per condition, we computed the error percentages and nine quantiles (.1 .2, …, .8, .9) of the RT distributions of correct responses. The weighted squared deviations of these error percentages and RT quantiles from the predicted ones were used in fitting the models by minimizing a root mean squared error. Details of the fitting procedure are described in Appendix B (OSM). After estimating the parameters, we took them for granted and computed RTs and error percentages for 1,000 samples with 4,223 trials per condition to derive “prediction intervals” in which 95% of samples with the same size as the ones actually collected would fall.

## Results

### The effect of irrelevant size

As opposed to Experiment 1, with spatial responses to the colors of stimuli, we expected an SSARC if it originated at response selection. In fact, with central stimulus location in the first part of the experiment there was a clear-cut SSARC effect: RTs in congruent trials with left responses to small stimuli and right responses to large stimuli were shorter (*M* = 357 ms; *SD* = 48) than in incongruent trials with left responses to large stimuli and right responses to small stimuli (*M* = 368 ms, *SD* = 58), *t*(89) = -3.596, *p* < .001, *d* = -0.379. Similarly error percentages were smaller in congruent trials (*M* = 2.546; *SD* = 3.571) than in incongruent trials (*M* = 3.102, *SD* = 4.308), but this difference was not significant, *t*(89) = -1.007, *p* = .317, *d* = -0.106.

With peripheral stimulus locations in the second part of the experiment there was also a clear-cut SSARC effect: RTs were shorter in congruent trials (*M* = 420 ms, *SD* = 62) than in incongruent trials (*M* = 429 ms, *SD* = 63). According to a two-way ANOVA with the within-participant factors Size Congruency and Location Congruency the main effect of Size Congruency was significant, *F*(1,89) = 19.946, *MSE* = 353.081, *p* < .001, ${\eta }_{p}^{2}$ = .183. Similarly error percentages (cf. Table 2) were smaller in congruent trials (*M* = 3.322, *SD* = 3.508) than in incongruent trials (*M* = 4.896, *SD* = 4.766). Again the main effect of Size Congruency was significant, *F*(1,89) = 26.111, *MSE* = 8.540, *p* < .001, ${\eta }_{p}^{2}$ = .227.

**Table 2:** Error percentages observed in Experiment 2 as a function of Location Congruency, and Size Congruency. Standard deviations are given in parentheses

		Size Congruency	
		Congruent	Incongruent
Location Congruency	Congruent	2.222 (2.876)	3.380 (3.650)
	Incongruent	4.421 (3.745)	6.412 (5.265)

### Interactions of irrelevant size and irrelevant location

**Fig. 4:**
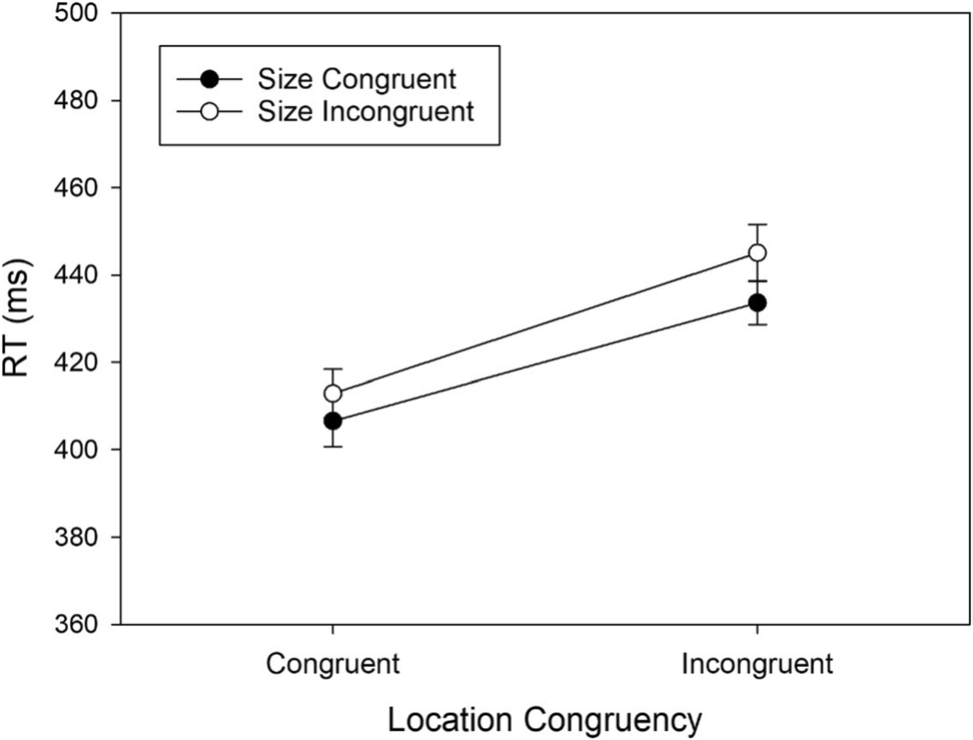
Reaction times (RTs) observed in Experiment 2 as a function of Location Congruency (Simon effect), and Size Congruency (SSARC effect). Error bars show 95% confidence intervals for within-subjects designs (Cousineau, 2017)

The RT means of the second part of the experiment are shown in Fig. 4. In addition to the significant effect of irrelevant Size (SSARC effect), there was a significant effect of irrelevant location (Simon effect), *F*(1,89) = 90.369, *MSE* = 871.552, *p* < .001, ${\eta }_{p}^{2}$ = .504. RTs were shorter in congruent trials with responses on the same side as the stimuli (*M* = 410 ms, *SD* = 62) than in incongruent trials with responses on the opposite side of the stimuli (*M* = 439 ms, *SD* = 65). Although Fig. 4 suggests a slightly larger effect of irrelevant size with incongruent irrelevant locations (or a slightly larger effect of irrelevant locations with incongruent irrelevant size), this interaction was not significant, *F*(1,89) = 1.904, *MSE* = 303.870, *p* = .171, ${\eta }_{p}^{2}$ = .021.

The error percentages of the second part of the experiment are shown in Table 2. Similar to the mean RTs, the effect of irrelevant location was significant, *F*(1,89) = 33.617, *MSE* = 18.318, *p* < .001, ${\eta }_{p}^{2}$ = .274. The error percentage was smaller in congruent trials (*M* = 2.801, *SD* = 1.473) than in incongruent trials (*M* = 5.417, *SD* = 4.664). Table 2 again suggests a slightly stronger effect of each irrelevant stimulus feature when the other was incongruent, but as for mean RTs the interaction was not significant, *F*(1,89) = 1.611, *MSE* = 9.700, *p* = .208, ${\eta }_{p}^{2}$ = .018.

Both for RTs and error rates there was an indication of the presence of an interaction of irrelevant size and irrelevant position, but in both cases it missed statistical significance in spite of the decent power of the present experiment. We noted that in both parts of the experiment the SSARC for RTs was rather small, 11 and 9 ms, respectively. For such small effects interactions might be hard to detect because they imply the detection of modulations by other variables, in our case by irrelevant location, in the range of only a few milliseconds. Therefore, we sought to examine interactions for a subgroup of participants with stronger SSARC effects. For this purpose, we split the sample at the median of the individual SSARC effects (6.65 ms) in the first part of the experiment with central stimulus locations, resulting in two subgroups with larger SSARC and smaller (or negative) SSARC effects.

**Fig. 5:**
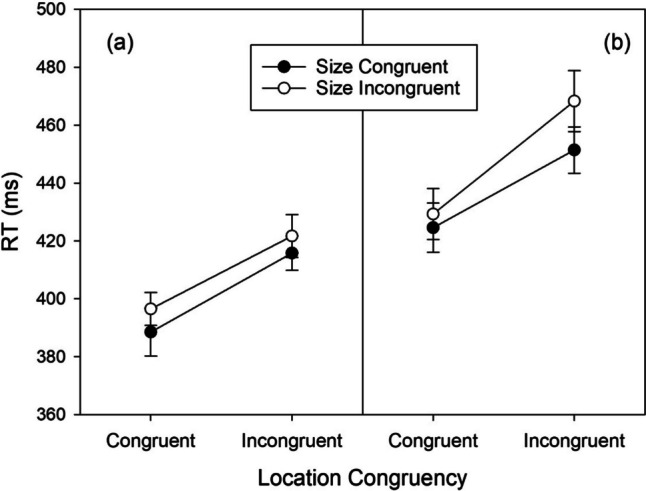
Reaction times (RTs) observed in Experiment 2 as a function of Location Congruency (Simon effect), and Size Congruency (SSARC effect), shown separately for two groups of participants with small (left panel [**a**]) and large (right panel [**b**]) SSARC effects in a control condition. Error bars show 95% confidence intervals for within-subjects designs (Cousineau, 2017)

Figure 5 shows the mean RTs of the two subgroups in the second part of the experiment with peripheral stimuli. A three-way ANOVA with the between-participant factor Group (below median vs. above median) and the within-participant factors Location Congruency and Size Congruency revealed a significant main effect of Group, *F*(1,88) = 9.169, *MSE* = 13,939.302, *p* = .003, ${\eta }_{p}^{2}$ = .094. In the subgroup with small SSARC effects RTs were considerably shorter than in the subgroup with large SSARC effects, 406 and 443 ms, respectively. Thus, the selection by size of the SSARC effects turned out to be also a selection by speed of responding.

In addition to the main effect of group there was a significant three-way interaction, *F*(1,88) = 4.036, *MSE* = 291.850, *p* = .048, ${\eta }_{p}^{2}$ = .044. From Fig. 5 it is evident that in the subgroup with small SSARC effects there was no interaction of Size Congruency and Location Congruency, but in the subgroup with large SSARC effects. Separate two-way ANOVAs for the two subgroups confirmed this. In the subgroup with small SSARC effects (cf. Fig. 5a) there was a significant Simon effect of 25 ms, *F*(1,44) = 47.907, *MSE* = 648.008, *p* < .001, ${\eta }_{p}^{2}$ = .521, and a significant SSARC effect of 7 ms, *F*(1,44) = 7.669, *MSE* = 282.438, *p* = .008, ${\eta }_{p}^{2}$ = .148, but the interaction was not significant, *F*(1,44) = 0.199, *MSE* = 247.316, *p* = .658, ${\eta }_{p}^{2}$ = .005. In the subgroup with large SSARC effects (cf. Fig. 5b) there was a significant Simon effect of 33 ms, *F*(1,44) = 44.588, *MSE* = 1,092,398, *p* < .001, ${\eta }_{p}^{2}$ = .503, and a significant SSARC effect of 11 ms, *F*(1,44) = 12.266, *MSE* = 424.300, *p* = .001, ${\eta }_{p}^{2}$ = .218. In addition, the two-way interaction was significant, *F*(1,44) = 4.936, *MSE* = 341.093, *p* = .031, ${\eta }_{p}^{2}$ = .101, with incongruency of the one irrelevant stimulus feature amplifying the detrimental effect of incongruency of the other irrelevant feature.

### Distributional analyses

**Fig. 6:**
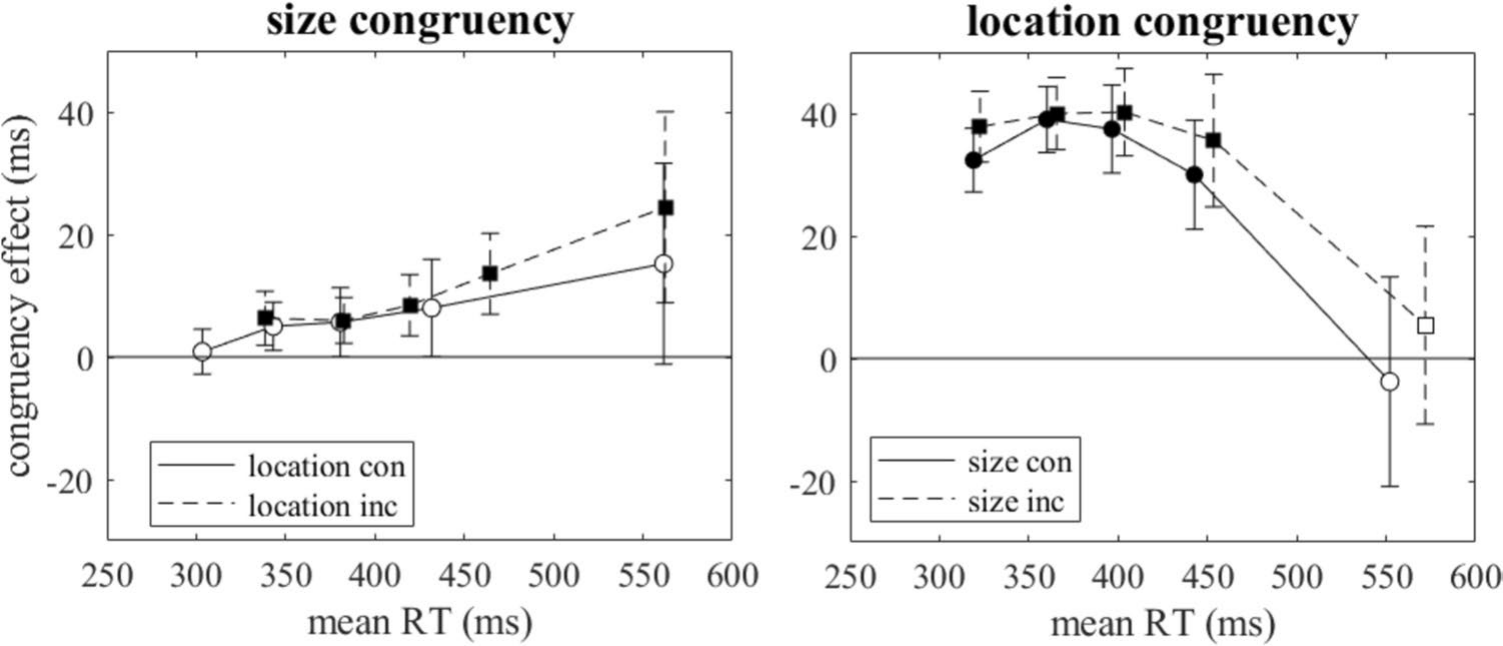
Size-congruency effect (SSARC effect) and location-congruency effect (Simon effect) at five quantiles of reaction time (RT) distributions as a function of the mean quantiles in congruent and incongruent conditions (delta plots), both for congruent and incongruent locations and stimulus sizes. Error bars show 95% confidence intervals. Filled symbols indicate significant differences between quantiles in congruent and incongruent conditions (after Bonferroni-Holm correction)

Analogous to Experiment 1, we analyzed the dynamics of the SSARC and the Simon effects. We computed five quantiles (.1, .3, .5, .7, .9) of the distributions of RTs of correct responses in size-congruent and incongruent conditions as well as location-congruent and incongruent conditions. Figure 6 shows the delta plots for size congruency (SSARC effect) and location congruency (Simon effect), that is, the differences between incongruent and congruent conditions at each quantile as a function of the mean quantiles in the two conditions.

As is evident from Fig. 6, the dynamics of SSARC and Simon effects were strikingly different. Whereas the SSARC effect was essentially zero at short RTs and increased at longer RTs, the Simon effect was present already at short RTs and basically declined as RTs increased, through the decline was preceded by a short period of increasing effect. Regarding the effects of congruency of the second irrelevant stimulus feature, that is, the interaction of location and size congruency, these were visible only at longer RTs. However, at none of the five quantiles the differences between the congruency effects observed with congruent and incongruent second irrelevant stimulus feature were statistically significant.

### Model-based analysis

Goodness-of-fit of the first-order and the second-order model was quite different. The root mean weighted squared deviations, which were minimized for parameter estimation, were 7.8 and 3.8, respectively. We also computed the chi-square values from the deviations between observed and predicted error probabilities and reaction-time probabilities in the bins defined by the observed quantiles, which were 43.9 and 10.6, respectively. The predicted means of RTs and error percentages in the four experimental conditions of the second part of the experiment are listed in Table 3. Note that the observed means were computed from the pooled RT distributions to which the models were fitted; tiny deviations from the means of individual mean RTs and error rates can result from individual variations in the rates of outliers and error responses. Although for RTs the predictions of the second-order model were generally closer to the observed means than the predictions of the first-order model, the differences were not as conspicuous as the difference between the goodness-of-fit criteria might lead one to expect.

**Table 3:** Observed and predicted mean reaction times (RTs; ms) and error percentages. Predictions are given by the first-order (1.order) and second-order (2.order) models together with the 95% prediction intervals (in italics, upper and lower values in each cell). Predicted values for which the prediction interval did not include the observed means are shown in bold

		RTs			Error Percentages		
		Observed	Predicted		Observed	Predicted	
Location	Size		1.order	2.order		1.order	2.order
Cong	Cong	406	*396* **399** *402*	*399* **402** *405*	2.2	*1.8* 2.2 *2.7*	*1.4* 1.8 *2.2*
Cong	Incong	413	*405* **408** *411*	*407* 410 *413*	3.5	*2.0* **2.5** *2.9*	*1.7* **2.1** *2.6*
Incong	Cong	434	*425* **428** *431*	*429* 432 *435*	4.5	*5.3* **6.0** *6.7*	*4.8* **5.4** *6.2*
Incong	Incong	444	*437* **440** *443*	*438* 441 *444*	6.5	*5.8* 6.6 *7.3*	*5.5* 6.2 *6.9*

Cong = congruent, incong = incongruent

**Fig. 7:**
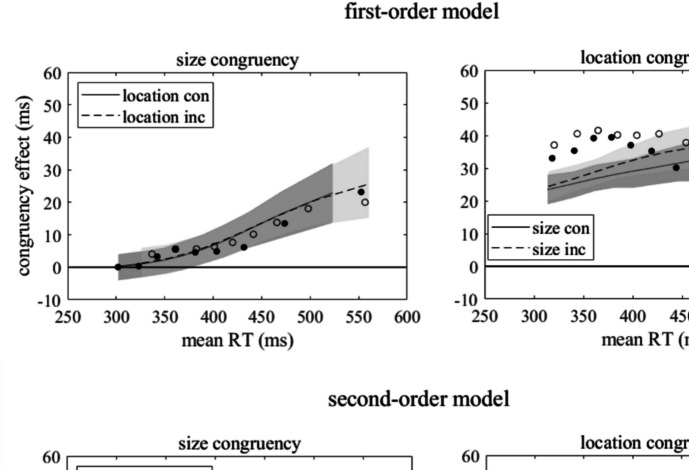
Simulated delta plots for the size-congruency effect (SSARC effect) with congruent and incongruent stimulus locations and the location-congruency effect (Simon effect) with congruent and incongruent stimulus sizes. Observed congruency effects (computed from pooled data) are shown by filled and open circles, predicted congruency effects by continuous and broken lines with shaded areas marking the 95% prediction intervals

The difference between the predictions of the two models becomes conspicuous as one examines the delta plots shown in Fig. 7. These delta plots were computed from nine quantiles of the combined individual distributions instead of only five quantiles as the delta plots in Fig. 6, which were computed from the individual distributions for which the trial number was much smaller. Across the range of RTs the SSARC effect (size-congruency effect) started at zero and increased. Both models reproduced this increase rather well, with the first-order model probably slightly better at the longest RTs. The Simon effect (location-congruency effect) was present already at the shortest reaction time and declined after a slight initial increase. The first-order model failed to reproduce this time course, whereas the second-order model did so in good approximation.

The estimated parameters of the second-order model are listed in Appendix C (OSM). Although these estimates may not be the only ones that allow a reasonable model fit (cf. Miletić et al., 2017; Turner et al., 2016), they suggest certain differences between the impacts of the irrelevant stimulus features. Most importantly, according to the estimated model parameters the impact of the task-irrelevant stimulus location led the impact of the relevant stimulus feature color, whereas the impact of the task-irrelevant stimulus size lagged the impact of the relevant stimulus feature. This difference accounts for the strong difference between the congruency effects at the short RTs. In addition, the initial impact of irrelevant size was slightly weaker than the initial impact of irrelevant location and declined considerably faster with the passage of time.

## Discussion

In Experiment 2, participants responded with spatially distinct (left-right) key-press responses to the color of a stimulus that also varied in stimulus size (part 1), or in stimulus size and stimulus location (part 2). Hence, we measured only the SSARC effect in part 1, and both the SSARC effect and the Simon effect in part 2 of the experiment. Experiment 2 revealed several notable results. First, we obtained comparable SSARC effects of irrelevant stimulus size both when stimulus location was constant (part 1) and when stimulus location varied unpredictably between left and right (part 2). Second, in part 2 of the experiment, the SSARC effect and the Simon effect appeared simultaneously. Third, the numerical trend towards an interaction between the SSARC and Simon effect was not significant when we considered the full sample of participants. Fourth, an exploratory analysis revealed a significant interaction between the SSARC and Simon effect in part 2 of the experiment for participants with a relatively large SSARC effect in part 1, whereas the interaction was absent for participants with a relatively small SSARC effect in part 1. Notably, the participants with larger SSARC effects in part 1 of the experiment were also slower than participants with smaller SSARC effects, replicating an already known impact of RTs on the size of the SSARC effect (Heuer et al., 2023). This result suggests that longer RTs and, as a consequence, larger SSARC effects are required to detect an interaction between the SSARC effect and the Simon effect. Finally, an LCA model assuming that irrelevant stimulus location and irrelevant stimulus size activate spatial response codes independently, and with different temporal offsets, was able to reproduce the observed pattern of experimental results with good precision. This model specifies how both the SSARC effect and the Simon effect can arise at the response-selection stage even without a (statistically significant) interaction.

## General discussion

The present study investigates the functional processing level on which the SSARC effect originates: Does the processing of size and space already interact at earlier, perceptual, levels of processing, or does the effect arise at later, response-related, levels of processing? In Experiment 1 we investigated whether the processing of stimulus size and the processing of stimulus location already interact at a perceptual level. We simultaneously varied the color, size, and location of a visual stimulus, and participants verbally responded to each feature dimension in a separate task. The critical task was color-naming because effects of S-R compatibility between stimulus size or stimulus location, respectively, and vocal color responses were not possible. In this task we failed to observe a significant congruency effect between stimulus size and stimulus location, suggesting that the processing of these stimulus features does not interact at early perceptual levels. In the location-naming task and the size-naming task, in contrast, we observed regular and reciprocal SSARC effects. In Experiment 2 we used additive-factors logic for investigating whether the SSARC effect originates at the response-selection stage or not. The interaction between the Simon effect and the SSARC effect was not significant when we tested it for the full sample, suggesting different loci at first sight. However, in an additional, exploratory analysis we observed a significant interaction for a subsample of participants with large/r SSARC effects in a control condition with central stimulus presentation, whereas the subsample of participants with small/er SSARC effects in the control condition did not show this interaction.

### The locus of the SSARC effect

The present experiments revealed three pieces of evidence for the notion that the processing of size interacts with the processing of space during response selection. First, there is the contrast between the results of Experiment 1 and Experiment 2: With left and right manual responses to the colors of stimuli which also vary in size (and location) there is a size-congruency effect (SSARC effect), whereas with naming the colors there is no such effect. Thus, the presence of spatial response features appears as a prerequisite for the effect of task-irrelevant stimulus size.

Second, there is the interaction of the SSARC effect and the Simon effect, which suggests a common stage of these effects according to the additive-factors logic of Sternberg (1969). As the Simon effect arises during response selection (Cespón et al., 2020; Lu & Proctor, 1995), this should be the common stage where the SSARC effect arises as well. In both the first part of the experiment with central stimulus locations, as in previous studies (e.g., Richter & Wühr, 2022; Wühr & Seegelke, 2018), and the second part with peripheral stimulus locations the mean SSARC effects were only small, 11 ms and 9 ms, respectively. In the full sample the critical interaction was numerically present, but statistically not significant. We hypothesized that this could be related to the small SSARC effects which allowed only a small range of modulations by the irrelevant stimulus locations and, vice versa, should produce only small modulations of the larger Simon effects. Therefore, we split the sample by the size of the individual SSARC effects observed with central stimuli in the first part of the experiment. For the subgroup with SSARC effects below the median there was no interaction of irrelevant size and location in the second part of the experiment, but for the subgroup with SSARC effects above the median the interaction was statistically significant.

Although the additional analysis of the two subgroups was motivated by the small SSARC effects, the difference between the subgroups in the second part of the experiment was more pronounced in mean RTs than in the size of the SSARC effects. In the first part of the experiment the size of the SSARC effect was significantly correlated with mean RT (*r* = .370, *p* < .001), and the difference between RTs persisted in the second part of the experiment. Thus, the probability for observing an interaction between the Simon effect and the SSARC effect seems to increase when overall RT increases. Perhaps, location- and size-driven processes of response activation must overlap for sufficient time to produce the interaction between the Simon effect and the SARC effect, and this in particular as the size-driven processes of response activation are absent at the fastest RTs and develop only gradually as RTs increase (Heuer et al., 2023; also Fig. 6).

With an overly simplified additive factors logic, according to which an interaction indicates that two variables affect the same stage of processing, whereas additive effects indicate that they affect different stages, one could entertain the hypothesis that Simon and SSARC effects arise at a common stage of processing for the subgroup that showed the interaction, but at different stages for the subgroup that did not show the interaction. However, even when the underlying assumptions of the additive factors logic are met and interactions between variables indicate effects on a common stage, additive effects do not necessarily indicate distinct stages (cf. Prinz, 1972; Sanders, 1980). Thus, the hypothesis of fundamental differences between the two subgroups, beyond the difference that the critical interaction can only be observed for the subsample of participants for which RTs are long enough for SSARC effects to be affected by the Simon effect (and vice versa) in a measurable way, but not in the other subgroup, is unjustified.

Third, the joint effects of irrelevant stimulus location and irrelevant stimulus size can fairly accurately be simulated by an LCA model according to which both irrelevant stimulus features drive activation of the correct or incorrect response, depending on their respective congruencies with response location. Whereas the impact of irrelevant location on response selection leads the impact of the relevant stimulus feature, the impact of irrelevant size lags. These different temporal offsets of irrelevant location and size are consistent with a previous analysis of separate effects of irrelevant location and irrelevant size (Heuer et al., 2023). In addition to the different temporal offsets, the impact of irrelevant size is slightly weaker than that of irrelevant location and dissipates more quickly. This model describes a mechanism by which both the Simon effect and the SSARC effect arise during response selection, without any assumption about interactions between the two irrelevant inputs during perceptual processing.

### Interactions of size and space subsequent to response selection?

Proponents of stage analysis have drawn a distinction between response selection and subsequent stages such as response programming or motor adjustment (Sanders, 1980, 1990), that is, between selection of an action and its activation. In fact, selection and activation of an action can be separated in time. This is evident from everyday life and many studies of delayed response tasks in humans and monkeys; it allows the experimental separation of movement specification and initiation even when specification is not yet completed (e.g., Ghez et al., 1997; Heuer et al., 1998) and has been added to a model of motor control (Bullock & Grossberg, 1988). However, in simple speeded binary decisions as in the present experiments, where individual stimulus features, such as two colors, are mapped to individual response features, such as left and right hand, perceptual discrimination is tightly linked to response initiation. Therefore we refer to the formal accumulators of the LCA model as “response codes,” implying that their activations reflect the corresponding activations of responses (cf. Heuer et al., 2023).

Tight links between perceptual discrimination, action selection, and initiation become apparent when one asks where in the central and peripheral nervous system systematic activations can be observed that mark the progress of a decision (often called “decision variable”) and thus correspond, for example, to the activation of response codes in a sequential-sampling model. Decision variables of this kind have been identified, for example, in the human EEG above the primary motor cortex (Servant et al., 2016) and in the frontal eye field of rhesus monkeys, with eye movements being initiated upon reaching a certain level of activity (Hanes & Schall, 1996). In a more straigthforward manner the tight link between selection and initiation of responses is indicated by the early (automatic) LRPs in the Simon task (see Leuthold, 2011) or the EMG activity without overt error response in incongruent trials (e.g., Burle et al., 2002), and further by the modulation of reflex gains depending on the forthcoming response (Selen et al, 2012). Thus, for tasks like the present ones, response selection and motor activation appear inseparable, whereas in delayed response tasks selection and initiation can be separated intentionally. However, even for tasks of the latter type we would expect the SSARC effect to arise during response selection. At least there is no obvious way in which a response selected in the face of conflicting information could still be affected by this conflict during initiation.

### Reciprocal SSARC effects

In the color-naming task of Experiment 1 we failed to observe evidence for interactions between the concurrent processing of stimulus location and stimulus size: RTs for processing congruent (small/left, large/right) and incongruent (small/right, large/left) stimuli did not differ significantly (and numerically in the wrong direction) when the stimulus dimensions location or size did not overlap with the response dimension (color). This result suggests that the significant congruency effects, which were observed in the location- and the size-naming tasks, were actually S-R compatibility effects that arose from stimulus size and stimulus location activating a congruent (location or size) response, and did not reflect size-location interactions at a perceptual level of processing. In the location-naming task, there was dimensional overlap between irrelevant stimulus size and the location-related response, producing a regular SSARC effect with ${\eta }_{p}^{2}$ = .083. In contrast, in the size-naming task, there was dimensional overlap between irrelevant stimulus location and the size-related response, producing a *reciprocal* SSARC effect with ${\eta }_{p}^{2}$ = .067 (Richter & Wühr, 2023) which, though, was not significant for the five quantiles of the RT distributions and may not be quite robust. The observation of this reciprocal SSARC effect is somewhat surprising anyway because, in our previous study, we observed a strong regular SSARC effect with vocal responses (*d* = 0.591), but no reciprocal SSARC effect when outlier datasets were excluded (*d* = 0.101). However, in a yet unpublished study, we observed both regular and reciprocal SSARC effects when location words instead of physical locations were used as stimuli in the reciprocal SSARC task (Richter & Wühr, 2024). The reasons for the discrepant findings concerning reciprocal SSARC effects are yet to be determined, but available evidence at least suggests that differences in stimulus mode can affect the size of reciprocal SSARC effects, and this may apply to other methodological differences as well.

## Conclusions

The results of Experiment 1 showed that the concurrent processing of stimulus size and stimulus location does not interact, and produce congruency effects, at a perceptual level of processing when both stimulus features are irrelevant for the task at hand. Moreover, the results of Experiment 2 suggest that the SSARC effect, like the Simon effect, arises at the response-selection stage. Together, this pattern of results suggests a genesis of the SSARC effect according to which the (relative) size of a visual stimulus is automatically processed, and the resulting size code activates a congruent location-related response code at the response-selection stage. In our view, these conclusions are compatible with a hypothesis about the origin of the SSARC effect positing that it arises from functional differences in using the two hands (cf. Wühr et al., 2024). According to this hypothesis, the higher strength of the dominant relative to the non-dominant hand (e.g., Bardo et al., 2021) creates the habit of grasping larger (and heavier) objects with the dominant hand, while grasping smaller (and lighter) objects with the non-dominant hand (cf. Wühr et al., 2024).

## Supplementary information

Below is the link to the electronic supplementary material.

Supplementary file1 (DOCX 185 KB)

## Data Availability

The raw data from both experiments are publicly available at the Mendeley Repository (10.17632/3v79sh9njf.1). Materials can be obtained from both authors upon request.

## Footnotes

1 The three-factorial design is different from, but equivalent to, the two-factorial design described in the preregistration of Experiment 1. In our preregistration, we had planned to combine the factors Stimulus Size and Stimulus Location to the higher-order factor *Congruency* with stimulus-feature combinations small/left and large/right forming the congruent condition, and the stimulus-feature combinations small/right, and large/left forming the incongruent condition. However, we now considered it more informative to analyze the two factors independently.

2 The three-way ANOVA produced the same pattern of results reported here when only the data from right-handed-participants were analyzed.

3 Experiment 2 was preregistered at the OSF (https://doi.org/10.17605/OSF.IO/6RKEC) on 16 April 2023, before data collection began.

4 After having finished the preregistration, we decided to include a condition in which stimulus location would remain constant (at screen center). This feature was added for three reasons. First, having a condition with central stimulus presentation allowed us to compare the SSARC effects of this sample in this condition to previous studies with the same features (e.g., Wühr & Seegelke, 2018). Second, having a condition with central stimulus presentation allowed us to assess how presenting size-variable stimuli at peripheral locations (in the main part of the experiment) would affect the SSARC effect. Third, having a condition with central stimulus presentation allowed us to perform an exploratory analysis, where we explored the effect of the size of SSARC effect, measured in a pure (control) condition, on the interaction between SSARC effect and Simon effect in a mixed (test) condition.

5 In the preregistration, we had planned to analyze the results in three-factorial ANOVAs with Stimulus Location, Stimulus Size, and Response Location as within-subjects factors. However, in this analysis, the important interaction between the SSARC effect and the Simon effect would not be reflected in the three-way interaction, as we would have expected, but in the two-way interaction of Stimulus Size and Response Location. Therefore, we decided to change the statistical model to a two-factorial model with Location Congruency, and Size Congruency as two within-subjects variables. This model is simpler, and the interesting interaction is directly reflected in the interaction between the two independent variables.

## Code availability

Programs for running the experiments, which were written in E-Prime 3.0, can be obtained from the first author upon request. Code used for modelling and simulating data can be obtained from the second author upon request.
