# Supplementary material for: Where does the processing of size meet the processing of space?
Source: Atten Percept Psychophys. 2024 Nov 12;87(4):1230–48. doi: 10.3758/s13414-024-02979-3 (PMC12058949; doi:10.3758/s13414-024-02979-3)
Supplement: Supplementary file 1 — Supplementary file1 (DOCX 185 KB) [file 13414_2024_2979_MOESM1_ESM.docx]

**Appendix A**

The Leaky, Competing Accumulator (LCA) model has been proposed by Usher and McClelland (2001). The extended LCA models for the SSARC effect and the Simon effect are described by Heuer et al. (2023, model B): The main additions are a second external input related to the irrelevant stimulus feature with an exponential decline during each decision and a temporal offset between the inputs from task-relevant and task-irrelevant stimulus features. Here we add a third external input related to the second irrelevant feature, and in addition to the exponential decline of the irrelevant inputs in the “first-order model” we made use of a more complex decline in the “second-order model”.

According to Usher and McClelland (2001, Eq. 3), the instantaneous activations Δa_c_(i) and Δa_e_(i) of the response codes for correct and incorrect responses during each time interval i are given by:

(1a) Δa_c_(i) = [ I(i) – λ a_c_(i) – β a_e_(i)] (Δt/τ) + ξ(i) √ (Δt/τ)

(1b) Δa_e_(i) = [ (1-I(i)) – λ a_e_(i) – β a_c_(i)] (Δt/τ) + ξ(i) √ (Δt/τ)

with self-inhibition gain λ, lateral-inhibition gain β, and Gaussian noise ξ(i) with zero mean and standard deviation σ_n_. The external inputs I(i) and 1-I(i) add to 1 (cf., Usher & McClelland, 2001, p.559). As a default we set I(i) = 0.5 so that they are identical for the correct- and error-response codes. The constraint on the sum of the external inputs results in a forward inhibition of the error-response code in addition to the lateral inhibition when I(i) >1 and thus 1-I(i)<0.

Beginning at initial values a_c_(0) = a_e_(0) = 0, the instantaneous activations of each response code (Eq. 1a, b) are cumulated with the constraint of non-negative response-code activations:

(2a) a_c_(i) = max [ 0, a_c_(i-1) + Δa_c_(i)]

(2b) a_e_(i) = max [ 0, a_e_(i-1) + Δa_e_(i)].

A response is initiated when the activation a_c_(i) or a_e_(i) reaches a threshold *θ*. A non-decision or residual time *R* is added to the time needed for the decision.

Previously (Wühr & Heuer, 2018; Heuer et al., 2023) we have extended the basic LCA model in two ways. First, we defined the total external input I(i) as the sum of two components (in addition to the default of 0.5): the time-invariant relevant input ΔI_rel_ and the time-varying irrelevant input g(t)*ΔI_irr._. Second, we added a temporal offset *D* between the relevant and irrelevant input with a uniform distribution (mean μ*_D_*, width w*_D_*). We defined the time at which the relevant input becomes available as t=0; with a sample value of *d*<0 the irrelevant input leads as it is typical for the impact of the irrelevant stimulus location, and with a *d*>0 the irrelevant input lags as it is typical for the impact of the irrelevant stimulus size. For *d* < 0 the external input is

(3a) I(t) = $\left\{ \begin{aligned} 0.5 + g\left( t \right)*\Delta I_{\mathrm{irr}} for t<0 \\ {0.5+\Delta I}_{\mathrm{rel}}+ g\left( t \right)*\Delta I_{\mathrm{irr}}\mathrm{for}t\geq0 \end{aligned} \right.$

and for *d* > 0 it is

(3b) I(t) = $\left\{ \begin{aligned} 0.5 + \Delta I_{\mathrm{rel}} for 0\leq t\leq d \\ 0.5 + \Delta I_{\mathrm{rel}}+ g\left( t \right)*\Delta I_{\mathrm{irr}}\mathrm{for}t>d \end{aligned} \right.$

Here we assume instantaneous external input not only from one task-irrelevant feature, but from both the irrelevant size (with initial strength $\Delta I_{\mathrm{irrS}}$) and the irrelevant location (with initial strength $\Delta I_{\mathrm{irrL}}$). In the first-order model these external inputs decline exponentially with the passage of time, $\Delta I_{\mathrm{irrS}}* e^{-(t-d_{S})/\delta_{S}}$ for irrelevant size and $\Delta I_{\mathrm{irrL}}* e^{-(t-d_{L})/\delta_{L}}$ for irrelevant location. The time constants $\delta_{S}$and $\delta_{L}$ can be different, allowing for slower or faster decline of the irrelevant inputs; $d_{S}$ and $d_{L}$ are sampled values of random variables *D_S_* and *D_L_*, of which the means (μ_DL_ and μ_DS_) and variabilities (widths w_DL_ and w_DS_ of the uniform distributions) can differ, allowing for different temporal offsets of the irrelevant inputs relative to the relevant one.

In the second-order model the exponential decline was replaced by the step response of a second-order high-pass filter: $\Delta I_{\mathrm{irrS}}* \left( 1-\frac{t-d_{S}}{\delta_{S}} \right)*e^{-(t-d_{S})/\delta_{S}}$ and $\Delta I_{\mathrm{irrL}}* \left( 1-\frac{{t-d}_{L}}{\delta_{L}} \right)* e^{-(t-d_{L})/\delta_{L}}$. In **Figure 2** the time courses of the irrelevant inputs for both models are illustrated for $\Delta I_{\mathrm{irrS}}=\Delta I_{\mathrm{irrL}}=1$, $d_{S}=d_{L}=0$, and $\delta_{S}=\delta_{L}=0.1.$

**Appendix B**

We fitted both the first-order and the second-order model to the pooled data of the second part of Experiment 2. For each model we estimated the parameters for the four conditions with location and size congruent and incongruent by minimizing the square root of the mean of the weighted squared deviations between predicted and observed relative error frequencies and quantiles of the distributions of the pooled reaction times of correct responses. Weights were set to one except for smaller weights for the clearly less reliable measures, namely the error probability and the highest two quantiles of the RT distributions. Reaction times of errors were neglected for fitting the model because error frequencies were small and mean error reaction times therefore quite unreliable.

More specifically, the minimized cost function was

(4) *C* = $1000 \sqrt{\frac{1}{m*8.75} A}$ with

$A = \sum_{j=1}^{m} \left[ {c_{0} \left( p_{j.ob}-p_{j.pr} \right)}^{2}+\sum_{k=1}^{9} c_{k}\left( P_{kj.ob}-P_{kj.pr} \right)^{2} \right]$,

where *j* = 1, …, m are the m conditions, *p* is the error probability, *P_k_* are the 9 quantiles of the pooled distributions of reaction times of correct responses, and *c_k_* are the weights (.5, 1, 1, 1, 1, 1, 1, 1, .75, .5 ) for the error probability and the quantiles in increasing order (8.75 is the sum of the weights for each condition). Subscripts *ob* and *pr* indicate the observed and predicted data, respectively. Multiplication by 1000 improves readability.

In fitting the models, we started each cost minimization with 1000 simulated trials per condition, which were increased up to 100,000 trials, using the MATLAB function *fminsearch*. The initial parameters were set somewhat intuitively to be not too far away from the final estimates. We used successive runs of the function with 75 iterations. The search ended when with 100,000 simulated trials per condition a criterion was reached that included changes of the parameters and the function value (parameters of *fminsearch* were TolX= 0.3 and TolFun=0.15). For the second-order model this criterion was not reached and the search was stopped after successive runs had brought no more improvement of the goodness-of-fit. The final costs were computed from an additional run of 100,000 simulated trials with the estimated parameters. From the final run of simulated trials we also computed χ^2^, based on the predicted and observed frequencies of errors and the predicted and observed frequencies of reaction-times of correct responses in the 10 bins defined by the 9 quantiles.

**Appendix C**

**Table C1** lists the parameter estimates for the better fitting second-order model. Most importantly, for the impact of stimulus location the mean temporal offset μ_DL_ was negative, that is, it led the impact of the relevant stimulus feature, whereas for the impact of stimulus size the mean temporal offset μ_DS_ was positive, that is, it lagged the impact of the relevant stimulus feature. This difference accounts for the strong difference between the location and size congruency effects at short RTs. In addition to the timing difference between the irrelevant stimulus features, the initial impact of irrelevant size, ΔI_irrS_, was slightly weaker than the initial impact of irrelevant location, ΔI_irrL_, and its decline with the passage of time was considerably faster as indicated by the difference between the time constants δ_S_ and δ_L_.

**Table C1:** Estimated parameters of the second-order model. The six parameters of the basic LCA model are listed first, followed by the four parameters for each of the external inputs related to the irrelevant stimulus location and size. Note that the differences between the four experimental conditions are captured only by the arithmetic signs of ΔI_irrL_ and ΔI_irrS_.

| Parameter | Description | Estimate |
| --- | --- | --- |
| λ | self-inhibition gain | .449 |
| β | lateral-inhibition gain | .270 |
| σ_n_ | standard deviation of noise | .405 |
| ΔI_rel_ | relevant external input | .386 |
| θ | response threshold | 1.157 |
| μ_R_ | residual time (s) | .205 |
| ΔI_irrL_ | irrelevant external input: location | ±.288 |
| δ_L_ | time constant for decline (s) | .150 |
| μ_DL_ | mean temporal offset (s) | -.052 |
| w_DL_ | width of temporal-offset distribution (s) | .144 |
| ΔI_irrS_ | irrelevant external input: size | ±.257 |
| δ_S_ | time constant for decline (s) | .057 |
| μ_DS_ | mean temporal offset (s) | +.112 |
| w_DS_ | width of temporal-offset distribution (s) | .145 |

**Appendix D**

Here we report the estimated model parameters (**Table D1**), the predicted means and error percentages (**Table D2**) and the delta plots (**Figure D1**) for the three tasks of Experiment 1 and the first part of Experiment 2 where only size congruency was varied. The analyses were analogous to those for the second part of Experiment 2, except that there was no impact of irrelevant stimulus location; for the color-naming task of Experiment 1 we also assumed no effect of irrelevant size.

**Table D1**: Estimated model parameters for the tasks of Experiment 1, and the SSARC effect measured in the first part of Experiment 2. The two final rows show goodness-of-fit measures.

|  |  | Experiment 1 | | |  | Experiment 2 |
| --- | --- | --- | --- | --- | --- | --- |
| Parameter |  | color naming | location naming | size naming |  | keypresses |
| λ |  | .066 | .199 | .217 |  | .346 |
| β |  | .253 | .214 | .242 |  | .251 |
| σ_n_ |  | .283 | .335 | .290 |  | .411 |
| ΔI_rel_ |  | .217 | .334 | .366 |  | .396 |
| θ |  | 1.498 | 1.249 | 1.351 |  | 1.050 |
| μ_R_ |  | .202 | .204 | .202 |  | .217 |
| ΔI_irrS_ |  | - | ±.087 | ±.068 |  | ±.323 |
| δ_S_ |  | - | .120 | .119 |  | .057 |
| μ_DS_ |  | - | +.190 | -.085 |  | +.144 |
| w_DS_ |  | - | .161 | .178 |  | .167 |
| C |  | 6.3 | 4.3 | 3.9 |  | 3.9 |
| χ^2^ |  | 21.2 | 19.5 | 12.1 |  | 11.7 |

**Table D2**: Observed and predicted mean reaction times and error percentages for the three tasks of Experiment 1 and the SSARC effect determined in the first part of Experiment 2. Predictions are given together with the 95% prediction intervals (in italics, left and right values in each cell). Predicted values for which the prediction interval did not include the observed means are in bold.

|  |  | Reaction Times | |  | Error Percentages | |
| --- | --- | --- | --- | --- | --- | --- |
| Task | Congruency | Observed | Predicted |  | Observed | Predicted |
| color naming | congruent | 451 | *449* 451 *454* |  | 0.7 | *1.1* **1.5** *1.9* |
|  | incongruent | 447 | *448* **451** *454* |  | 0.5 | *1.1* **1.5** *1.9* |
| location naming | congruent | 384 | *383* 385 *387* |  | 0.4 | *0.4* 0.7 *1.0* |
|  | incongruent | 389 | *387* 389 *391* |  | 0.9 | *0.6* 0.9 *1.3* |
| size naming | congruent | 445 | *444* 446 *449* |  | 0.6 | *0.7* **1.0** *1.3* |
|  | incongruent | 450 | *449* 451 *454* |  | 0.9 | *0.9* 1.3 *1.7* |
| keypresses | congruent | 357 | *357* 360 *363* |  | 2.6 | *1.5* 2.0 *2.6* |
|  | incongruent | 368 | *363* 366 *369* |  | 3.1 | *2.2* 2.9 *3.7* |

In Table D1 the goodness-of-fit criteria are given in addition to the parameter estimates. They were poorest for the color-naming task, for which also mean RTs and error percentages tended to be outside the 95% ranges of simulated samples (Table D2). For the SSARC effects observed with vocal responses in the location-naming task of Experiment 1 and the with manual responses in the first part of Experiment 2, the modeling suggests some differences: for manual responses the initial impact of the irrelevant size (ΔI_irrS_) was stronger, .323 vs .087, and less delayed relative to the impact of the relevant stimulus feature (μ_DS_, 144 vs 190 ms), but dissipated faster as indicated by the shorter time constant δ_S_, 57 vs 120 ms. These parametric differences are reflected in the differences between the delta plots of Figure D1: with manual keypresses the congruency effects starts to rise at shorter RTs than with vocal responses, and the increase seems to be steeper.


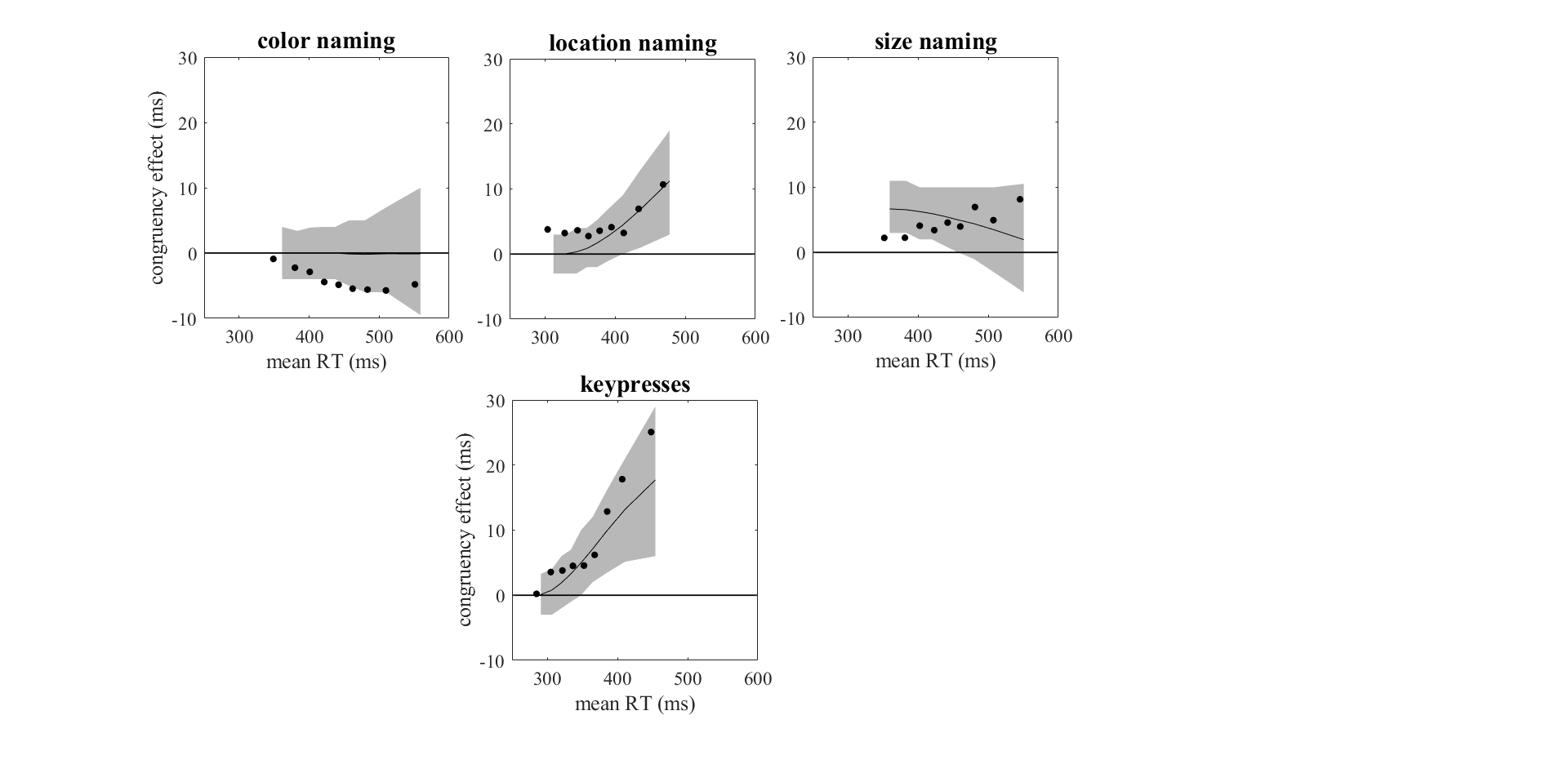


**Figure D1**: Simulated delta plots for the congruency effects observed in all three tasks of Experiment 1 with vocal responses and the first part of Experiment 2 with manual responses. Observed congruency effects (computed from pooled data) are shown by filled circles, predicted congruency effects by continuous lines with shaded areas marking the 95% prediction intervals.
